# Cell-specific toxicity of short-term JUUL aerosol exposure to human bronchial epithelial cells and murine macrophages exposed at the air–liquid interface

**DOI:** 10.1186/s12931-020-01539-1

**Published:** 2020-10-17

**Authors:** Rakeysha Pinkston, Hasan Zaman, Ekhtear Hossain, Arthur L. Penn, Alexandra Noël

**Affiliations:** 1 Department of Environmental Toxicology, College of Sciences and Engineering, Southern University and A&M College, Baton Rouge, LA 70813 USA; 2 Department of Comparative Biomedical Sciences, School of Veterinary Medicine, Louisiana State University, 1909 Skip Bertman Drive, Baton Rouge, LA 70803 USA

**Keywords:** JUUL, Electronic nicotine delivery system (ENDS), Electronic-cigarettes, Vaping, Air–liquid interface (ALI)

## Abstract

**Backgroud:**

JUUL, an electronic nicotine delivery system (ENDS), which first appeared on the US market in 2015, controled more than 75% of the US ENDS sales in 2018. JUUL-type devices are currently the most commonly used form of ENDS among youth in the US. In contrast to free-base nicotine contained in cigarettes and other ENDS, JUUL contains high levels of nicotine salt (35 or 59 mg/mL), whose cellular and molecular effects on lung cells are largely unknown. In the present study, we evaluated the in vitro toxicity of JUUL crème brûlée-flavored aerosols on 2 types of human bronchial epithelial cell lines (BEAS-2B, H292) and a murine macrophage cell line (RAW 264.7).

**Methods:**

Human lung epithelial cells and murine macrophages were exposed to JUUL crème brûlée-flavored aerosols at the air–liquid interface (ALI) for 1-h followed by a 24-h recovery period. Membrane integrity, cytotoxicity, extracellular release of nitrogen species and reactive oxygen species, cellular morphology and gene expression were assessed.

**Results:**

Crème brûlée-flavored aerosol contained elevated concentrations of benzoic acid (86.9 μg/puff), a well-established respiratory irritant. In BEAS-2B cells, crème brûlée-flavored aerosol decreased cell viability (≥ 50%) and increased nitric oxide (NO) production (≥ 30%), as well as *iNOS* gene expression. Crème brûlée-flavored aerosol did not affect the viability of either H292 cells or RAW macrophages, but increased the production of reactive oxygen species (ROS) by ≥ 20% in both cell types. While crème brûlée-flavored aerosol did not alter NO levels in H292 cells, RAW macrophages exposed to crème brûlée-flavored aerosol displayed decreased NO (≥ 50%) and down-regulation of the *iNOS* gene, possibly due to increased ROS. Additionally, crème brûlée-flavored aerosol dysregulated the expression of several genes related to biotransformation, inflammation and airway remodeling, including *CYP1A1*, *IL-6*, and *MMP12* in all 3 cell lines.

**Conclusion:**

Our results indicate that crème brûlée-flavored aerosol causes cell-specific toxicity to lung cells. This study contributes to providing scientific evidence towards regulation of nicotine salt-based products.

## Background

Since their entry into the US market in 2007, electronic nicotine delivery system (ENDS) devices, popularly called electronic-cigarettes (e-cigs), have lowered the incidence of active tobacco smoking [1]. The battery-operated ENDS devices, designed to deliver an inhalable heated aerosolized mixture of nicotine, flavoring compounds, and humectants [propylene glycol (PG) and vegetable glycerin (VG)] are marketed as, and believed by many to be, a safer alternative to cigarette smoking [2]. E-cig devices were formally targeted to adult smokers attempting to quit smoking; however, e-cigs have quickly gained immense popularity among adolescents and young adults, making these devices the most popular form of tobacco product usage within this demographic [1]. Moreover, ENDS products are more attractive to youth (< 18 years of age) compared to cigarettes at least in part due to targeted marketing and availability of a substantial assortment of flavored e-liquids [3–6]. According to the Centers for Disease Control and Prevention (CDC), e-cig usage among youth has increased to more than 5 million individuals in the US as of 2019 [7, 8], in addition to about 8 million adult users [9]. This striking increase in ENDS usage among youth and young adults has been suggested to be due in large part to the popularity of JUUL devices [10].

JUUL is a leading e-cig brand in the US, and in 2018, controlled more than 70% of the e-cig market shares. Even though JUUL only began to be marketed in 2015, in 2018 it was the most popular brand used among pre-teens, teens and young adults [11]. The concentration and form of nicotine could be one reason for the popularity of JUUL compared to other e-cig brands. The JUUL device, similar in size and shape to a memory stick, is uniquely designed to utilize disposable pods containing e-liquids consisting of benzoic acid and high levels of nicotine in the form of nicotine salts, labeled as 3% or 5% of nicotine (35 or 59 mg/mL) [12]. The latter pod liquid concentration is equivalent to the nicotine content of 1 full pack of filtered unburned cigarettes (10–15 mg nicotine/unburned cigarette; 1–2 mg nicotine absorbed) [13, 14]. JUUL pod’s liquid composition allows the nicotine salts to be heated at lower temperatures compared to other ENDS devices (e.g. 3rd generation e-cig device), which allows high levels of nicotine to be inhaled more easily, with minimized discomfort and optimal absorption [15–20]. This gives rise to a transfer of both freebase nicotine and the constituent acid (in this case benzoic acid) to the JUUL user [12, 15–17], in contrast to other types of e-cigs that use “freebase nicotine” at lower concentrations (3–36 mg/mL) [18], because high freebase concentrations can provide an unpleasant feeling to the throat and lungs [1, 19, 20].

Many young ENDS users do not realize that the majority of e-cig products contain nicotine. A recent study, published in BMJ’s Tobacco Control journal, found that although many young users are aware of JUUL, they are not necessarily aware of the high nicotine content [21]. It also was recently reported that 15–17 year-olds are 16 times more likely than adults between the ages of 25–34 years to use JUUL [22]. This increases the possibility that teenage JUUL users will develop a life-long addiction to nicotine, and switch to a more complex e-cig product or even turn to traditional tobacco products [1, 2]. If continued use of JUUL or other e-cig devices were shown to cause long-term respiratory effects, widespread e-cig and JUUL usage among teens and young adults would become a major public health concern [1, 23, 24].

Humectants and flavoring additives used in e-liquids are “generally regarded as safe” (GRAS) in food and cosmetic items [25]; however, the safety of flavoring additives when inhaled has not been thoroughly established [26]. For instance, several studies showed that the cytotoxic effects of ENDS products are linked to the presence of multiple favoring chemicals [26–31]. Indeed, flavoring chemicals have been reported to cause lung toxicity and respiratory conditions, including asthma, when inhaled, thus posing a potential threat to the respiratory health of users [32–34]. Common flavoring agents in e-liquids include aldehydes, such as vanillin, and alcohols, such as ethyl maltol, which have been identified in JUUL pod flavored liquids, including crème brûlée and mango [31, 35, 36]. These chemicals have been shown to be cytotoxic, cause respiratory irritation [30, 31, 35], and reduced lung function [37]. Flavoring additives chemically react with humectants (PG/VG) to form aldehyde acetals (e.g., vanillin PG/VG acetal), which activate pro-inflammatory respiratory irritant receptors [38, 39]. Moreover, these flavoring chemicals transfer from the liquid phase to the aerosol phase at a 39–79% efficiency rate [31, 35]. Vanillin PG acetal and vanillin VG acetals also were identified in JUUL crème brûlée pod liquids and transferred to the aerosol at a rate of about 68% (0.8 ± 0.04 mg/puff) and 59% (7.9 ± 0.8 mg/puff), respectively [35]. In addition to the formation of harmful acetals, humectants, when heated by an ENDS device, have been shown to form toxic carbonyl compounds [40–45], some of which, including formaldehyde, are carcinogenic and have been suggested to play a role in several respiratory diseases commonly seen in cigarette smokers [45, 46]. Another concern is that base ingredients of e-cig liquids (PG, VG) could be respiratory irritants and be harmful to the lungs, leading to airway obstruction and inflammation [47, 48]. While benzoic acid is often used as a food preservative, in inhalable form, it is a well-known respiratory irritant that can cause coughing and sore throat if exposure continues [49]. Further, heated and aerosolized e-liquid from any ENDS device can produce fine and ultrafine particles that contain polyaromatic hydrocarbons, carbonyls, aldehydes and volatile organic compounds, that are similar to the chemicals that are found in cigarette smoke, although in lower amounts, and that are sufficiently small to reach the lower airways [50, 51].

In vitro studies have shown that flavored e-cig aerosols can induce inflammation and oxidative stress, both with and without nicotine [26, 29, 30, 52, 53], although some studies have yielded conflicting results [54, 55]. A study by Omaiye et al. [31] investigated the flavoring and nicotine concentrations in various flavored JUUL pods including crème brûlée. They also evelauated the cytotoxicity of JUUL pod aerosol extracts on BEAS-2B cells using submerged culture conditions. They observed that JUUL crème brûlée-flavored pods contained about 60.9 mg/mL of nicotine along with high levels of vanillin (> 1 mg/mL) and ethyl maltol (0.65 mg/mL) in the aerosol extract. They also observed that JUUL crème brûlée is cytoxic and correlated with nicotine, ethyl maltol and total chemical concentrations within the aerosol extract, which suggests that both nicotine salt and flavorings may affect the health of lung cells. Another study by Muthamage and colleagues [30] investigated the effects of JUUL pod and JUUL-like pod aerosols on the human monocyte cell line U937. The cells were exposed to JUUL aerosol for 22 min under conventional submerged culture conditions. It was showed that monocytes treated with café latte- and classic menthol-flavored JUUL-like pods displayed cytotoxicity. The same group previously reported that ortho-vanillin (a common e-liquid flavoring chemical) reduced cell viability in U937 cells [29]. They also found that flavoring mixtures are more cytotoxic, and thus detrimental, than a single or more simple e-liquid flavor. Further, human lung epithelial cell lines (BEAS-2B and H292) and human primary lung fibroblasts (HLF-1) were used to investigate the effects of ortho-vanillin and maltol, two flavoring chemicals [28]. Although no significant decline in cell viability (80–85% post-treatment) was observed, maltol and ortho-vanillin, each at 1 mM, significantly increased the release of IL-8 in BEAS-2B and HLF cells compared to the untreated controls, whereas H292 cells showed no response. This suggested that ortho-vanillin and maltol may trigger an inflammatory response through the production of the inflammatory cytokine IL-8 in BEAS-2B and HLF-1 cells, but not in H292 cells [28]. Overall, currently, a very limited number of studies have investigated the effects of JUUL aerosols in vitro [30, 31]. Furthermore, no study has investigated the cellular and molecular effects of JUUL aerosols on cells exposed at the air–liquid interface (ALI).

To date, most in vitro studies investigating the toxicity of e-cig liquids and aerosols have been conducted using traditional submerged cell culture conditions, which do not take into consideration the physiological state of the lung, including pseudostratified epithelium, the presence of ciliated cells, or of immune cells, such as macrophages, which altogether act as a protective barrier against environmental insults, including inhaled toxicants, pathogens and allergens [56, 57]. ENDS aerosols, including JUUL aerosols, contain several compounds that may be insoluble in cell culture media. This may affect the dosage and potency of the aerosol under submerged in vitro conditions and ultimately result in producing non-translational biological outcomes [58]. Therefore, increased attention is being focused on using in vitro ALI test systems as a more physiologically relevant way of delivering and assessing toxicity of inhaled aerosols, by exposing the cells directly to the whole aerosols without using extracts or flavoring chemical that were not processed through an ENDS device [55, 58, 59]. With ALI, the apical surface of the cells is exposed only to air or the test atmosphere, while the basal surface of the culture system is in contact with the cell culture medium. This feature mimics the natural state of cells found in the airway and helps drive cellular differentiation.

Since scientific evidence regarding JUUL aerosol cytotoxicity is limited, the present study was designed to use an ALI system to investigate the effects of crème brûlée-flavored JUUL aerosol on human bronchial epithelial cell lines (BEAS-2B and H292) and on a mouse macrophages cell line (RAW 246.7). We selected to study the effects of JUUL crème brûlée, as it was one of JUUL’s top 3 ‘most’ popular flavors in 2019. With the increasing popularity of JUUL and JUUL-like devices among teens and young adults, it is clearly important to investigate cellular effects of JUUL aerosols to help guide future regulations of nicotine salt based e-cig products.

## Methods

### JUUL liquid and aerosol chemical analysis

The crème brûlée pods (5% nicotine) were purchased from JUUL labs (San Francisco, CA). The pod liquid was analyzed independently for nicotine and propylene glycol content by gas and liquid chromatography (GC/LC) techniques (Bureau Veritas, Buffalo, NY). JUUL aerosol chemical characterization for carbonyls was performed by collecting 40 puffs of JUUL aerosol following a protocol as described previously [60]. In brief, The JUUL device was connected to a programmable parastaltic pump (Cole-Parmer Masterflex L/S) with 1 in. diameter tygon tubing, followed by direct connection of a holder containing the Cambridge filter pad for direct capture of the JUUL aerosol. Samples were collected at a loading regimen of 1 L/min. The quantification of nicotine was performed by GC with a flame ionization detector (GC-FID), and of carbonyls by the EPA method TO-11A, based on high performance liquid chromatography [(HPLC), Enthalpy Analytical, LLC, Durham, NC]. In brief, to collect the JUUL aerosol for organic acid analysis, the JUUL device was connected to the peristaltic pump followed by a direct connection to a fritted glass impinger containing 30 mL acidified 2,4-dinitrophenylhydrazine aerosol trapping solution. The analysis of organic acids was conducted by ion chromatography. All samples were collected on site at the Inhalation Research Facility at Louisiana State University (LSU), School of Veterinary Medicine (SVM) and were shipped overnight on dry ice to Enthalpy Analytical, LLC.

### Cell lines and culture conditions

BEAS-2B cells (human bronchial epithelial cell line; ATCC CRL-9609), H292 cells (human bronchial epithelial cell line; ATCC CRL-1848), and RAW 246.7 cells (murine macrophage cell line; ATCC TIB-71), have a standard cell population doubling time of 24–48 h according to the provider. Cells were grown and maintained as a monolayer in T-75 tissue culture flasks until used for study. BEAS-2B cells were maintained in DMEM medium with 10% fetal calf serum, 100 U/mL penicillin, 100 μg/mL streptomycin. H292 and RAW 246.7 cells were maintained in RPMI-1640 medium with 10% fetal calf serum, 100 U/mL penicillin, 100 μg/mL streptomycin. The cells were incubated at 37 °C, in a 100% humidified atmosphere containing 5% CO_2_. Cells between passages 6 and 13 were used.

Once a sufficient number of cells were obtained, BEAS-2B or H292 cells were seeded onto 24 mm transwell with a 0.4-μm pore size polyester membrane insert (Catalog #3450, Corning Incorporated, Corning, NY) (4.2 cm^2^ cell culture area/insert) in 6-well plates, at a seeding density of ~ 2.5 × 10^4^ cells/insert. Media on apical and basolateral sides of the membrane was changed every 2–3 days. After attaining confluency around day 21 to generate a pseudostratified epithelium, apical cell media was removed from the cells to create the air–liquid interface condition of the cells, which induces epithelial stratification. The following day, cells were exposed to JUUL aerosols.

Raw 246.7 macrophages also were seeded on Corning transwell inserts at a density of ~ 1.5 × 10^6^ cells/insert. Since macrophages, unlike epithelial cells, do not undergo differentiation, on the day after seeding, apical media was removed, and macrophages were exposed to JUUL aerosols.

### JUUL aerosol generation and ALI cell exposures

A JUUL rechargeable pod-based device (JUUL Labs) was used for aerosol generation and cell exposures. To generate the crème brûlée-flavored JUUL aerosols, the JUUL device was connected to a programmable peristaltic pump (Cole-Parmer Masterflex L/S) with 1 in. diameter tygon tubing, followed by connection to a Vitrocell ALI exposure system. We used a customized ALI exposure system from Vitrocell Systems GMBH (Waldkirch, Germany) that enables direct exposure of cells to various aerosols. Our customized ALI system is composed of a Vitrocell 6/4 stainless steel exposure module for 4 × 6 well/24 mm diameter inserts, which is connected to a distribution system for the Vitrocell 6 modules. This ALI system is comprised of two exposure chambers, arranged in parallel, in which the cell inserts were exposed at the ALI to JUUL aerosols or to medical grade compressed air. The chamber used for JUUL exposures contained 4 wells. Three wells were dedicated to exposure of cells grown on inserts to JUUL aerosols, and one well for assessment of real-time aerosol deposition by a Quartz Cristal Microbalance (QCM; Vitrocell). The chamber used for air controls contained 3 wells in which cells were exposed to medical grade compressed air. Immediately before the exposure, the transwell inserts were transferred to the Vitrocell exposure system. The wells of the exposure device contained 7 mL of complete culture media on the basolateral side to maintain the cells during experiments. The chambers were connected to a water bath to maintain the cell temperature at 37 °C. Cells were exposed to crème brûlée-flavored JUUL aerosol or medical grade compressed air at the ALI following a standard vaping topography profile of 5 s duration every 30 s [61–63] for 1 h to mimic actual JUUL use patterns. JUUL aerosol was exposed to the cells at a flow rate of 0.36 LPM and diluted with medical grade compressed air at a flow rate of 0.5 LPM. Exposures to JUUL aerosol (n = 3 transwell inserts) and medical grade compressed air (n = 3 transwell inserts) were conducted in parallel. Following the exposure, cells were removed from the ALI chamber and placed back into the 6-well plates and then incubated at 37 °C for 24 h before cells were collected for analysis. Data represent a representative experiment (from two or three independent experiments).

### Scanning electron microscopy (SEM) analysis

SEM was performed to observe the morphological appearance of the cells after exposure to JUUL aerosol. Twenty-four hours after JUUL ALI exposures, the transwell membrane inserts (apical and basolateral surface) were washed twice with PBS followed by fixation with 1.25% (v/v) glutaraldehyde and 2% formaldehyde in 0.1 M sodium cacodylate buffer, pH 7.4 for 1 h at room temperature. The apical and basal chambers were washed three times with 0.1 M sodium cacodylate with 5% sucrose for 30 min, followed by post-fixed in 1% phosphate-buffered osmium tetroxide for 1 h at room temperature; then rinsed again 3 times × 10 min in 0.1 M PBS. The membranes were detached from the insert and dehydrated through increasing concentrations of ethanol. Samples were further dehydrated by incubation in hexamethyldisilizane before being placed in a desiccator overnight. The membranes were cut from the inserts and mounted on aluminum stubs before analysis on a FEI Quanta 3D scanning electron microscope. Images were taken at an accelerating voltage of 5 kV.

### Cell viability

Cell viability was evaluated by Trypan blue staining 24 h after exposure. Cells were stained with 10 μL of 1:1 trypan blue solution for 1 min. Viability was evaluated using a TC10 counting slide (Catalog #1450015, Bio-Rad Laboratories, Hercules, CA), which was placed in the TC20 automated cell counter (Catalog #1450102, Bio-Rad Laboratories, Hercules, CA). Duplicate readings of each sample were taken.

### Transepithelial electrical resistance (TEER)

On the day of cell collection, for H292 and BEAS-2B cells, trans-epithelial electrical resistance (TEER) was measured using a 4 mm chopstick electrode attached to the Millicell-ERS voltohmmeter (Catalog # MERS00002, Millipore, Burlington, MA). Electrodes were sterilized in ethanol and equilibrated in PBS prior to taking resistance measurements. Fresh media was added apically and basolaterally to the transwell membrane in a new 6-well plate. The electrodes were placed into the culture system (one in the apical compartment and one in the basolateral compartment) and the resistance across the transwell membrane was measured. The average of three measurements per transwell insert was determined. A blank measurement was taken using an insert without cells and was subtracted from each reading average. The resistance (Ω) was then multiplied by the surface area of the transwell membrane (0.45 cm^2^) to provide a final value (Ω cm^2^). After the measurement, the apical medium was discarded to restore ALI conditions.

### Lactate dehydrogenase (LDH) cytotoxicity/cell integrity assay

Extracellular release of LDH was measured in the basolateral culture media at 24 h post-exposure to JUUL aerosol. The assay was carried out according to manufacturer's instruction (CyQuant, LDH cytotoxicity assay kit, Catalog # C20300, Invitrogen, Thermo Fisher Scientific, Waltham, MA). Cytotoxicity of both air control- and JUUL-exposed samples were compared with cytotoxicity of positive control (manufacturer provided) samples. LDH was quantified spectrophotometrically (TECAN infinite 2000) by measuring at 490 nm, with 630 nm as reference wavelength. For each sample, the absorbance was normalized to the total cell count. The absorbance values for the air control groups were set at 100%. All samples were run in triplicate.

### Reactive oxygen species (ROS) quantification assay

The fluorogenic reagent OxyBURSTGreen H2HFF-BSA (Catalog # D2935, Invitrogen, Thermo Fisher Scientific, Waltham, MA) was used to detect extracellular ROS release in the basolateral culture media at 24 h post-exposure to the JUUL aerosol. The assay was carried out according to the manufacturer’s instructions. Fluorescence was measured spectrophotometrically (TECAN infinite 2000; excitation: 488 nm, emission: 530 nm). For each sample, the fluorescence was normalized to the total cell count. The fluorescence values for the air control groups were set at 100%. All samples were run in triplicate.

### Griess assay

Extracellular nitric oxide (NO) release from all cell lines used was quantified by photometrical detection of NO with a Griess reagent kit (Catalog #30100, Biotium, Fremont, CA). 24-h after the JUUL-aerosol exposure, the media was collected, and the content of NO was measured in accordance with the manufacturer’s protocol. The optical density (OD) of each sample was measured at 548 nm spectrophotometrically (TECAN infinite 2000). For each sample, the absorbance was normalized to the total cell count. The absorbance values for the air control groups were set at 100%. All samples were run in triplicate.

### RNA isolation and real-time quantitative reverse-transcriptase PCR analyses

Collected cells from all 3 cell lines were pelleted and total RNA isolation was performed using the RNeasy Plus Mini Kit (Cat # 74136, Qiagen, USA). Quantification of mRNA was done by spectrophotometry (260/280 nm ratio, NanoDrop 1000, Thermo Scientific). The high capacity RNA-to-cDNA kit (Cat # 4387406, Applied Biosystems, USA) was used for reverse transcription. Primer/probe sets were obtained as Taqman pre-developed assay reagents (concentrated and pre-optimized mix of primers and FAM-labeled Taqman probe) from Applied Biosystems (University Park, IL). SYBR green gene primer sets with gene specific forward and reverse primers were used for the human cell lines. The specific primers that were designed for gene amplification are listed and described in Additional file 1: Table S1. Reaction volumes were 25 μL, and 40 reaction cycles were run for each gene in an Applied Biosystems 7300 Real-Time PCR System. We used the comparative cycle threshold (ΔΔC_T_) method to determine relative gene expression, with each gene normalized to either *β-ACTIN* (human cells) or hypoxanthine guanine phosphoribosyltransferase (*Hprt1*) (murine cells) expression. Results are reported as fold change over control [(2^−ΔΔCT^)]. A fold-change > ± 1.5 with a p-value < 0.05 was considered significant.

### Statistical analysis

All biological outcomes were analyzed by the Student *t*-test for pairwise comparisons. Results are presented as mean ± standard error of the mean (SEM). Results were considered statistically significance at p < 0.05. We carried-out statistical analyses with the GraphPad Prism 8 Software (GraphPad Software, San Diego, CA).

## Results

### Chemical characterization of JUUL crème brûlée-flavored liquid and aerosol

Before studying the cellular and molecular effects of JUUL crème brûlée-flavored aerosol, we investigated the composition of the pod’s liquid (nicotine and humectant components) and that of the subsequent aerosol (nicotine, humectants, carbonyls and organic acids). All testing was performed through third party chemical testing companies. JUUL lists their pod humectant ratio as 30/60 PG/VG, with 59 mg/mL of nicotine salt. Our results were that the JUUL crème brûlée-flavored pod contains 29.6% of propylene glycol and ~ 52 mg/mL of nicotine, which is very close to levels advertised, and also coincides with levels of nicotine previously identified in JUUL pods [31] (Table 1). JUUL aerosols contained nicotine (0.131 mg/puff), PG (0.680 mg/puff) and VG (1.81 mg/puff) (Table 1). Regarding carbonyl compounds, JUUL aerosol contained low levels of formaldehyde (0.053 µg/puff), along with trace amounts of acrolein, acetaldehyde and diacetyl (Table 1). We also tested for organic acids, specifically benzoic acid, because JUUL pod liquids are advertised to contain benzoic acid as part of its patented nicotine salt formulation. We found that JUUL crème brûlée-flavored aerosol contains high levels of benzoic acid (86.9 µg/puff) (Table 1). Collectively, our results demonstrate that crème brûlée-flavored JUUL aerosol contains relatively low levels of carbonyls, but high levels of benzoic acid.

**Table 1:** JUUL crème-brûlée-flavored pod and aerosol chemical analysis

Chemical	Quantity
Pod chemical analysis	
Nicotine	52.5 mg/mL
Glycerin	29.60% (v/v)
Aerosol chemical analysis (nicotine, carbonyls and organic acids)	
Nicotine	0.131 mg/puff
Propylene Glycol	0.680 mg/puff
Glycerin	1.81 mg/puff
Acrolein	< LOQ (< 0.042 µg/puff)
Acetaldehyde	< LOQ (< 0.042 µg/puff)
Diacetyl	< LOQ (< 0.013 µg/puff)
Formaldehyde	0.053 µg/puff
Benzoic acid	86.9 µg/puff

*LOQ* limit of quantification

### JUUL crème brûlée-flavored aerosol alters cell morphology and induces cytotoxic responses in BEAS-2B cells

**Fig. 1:**
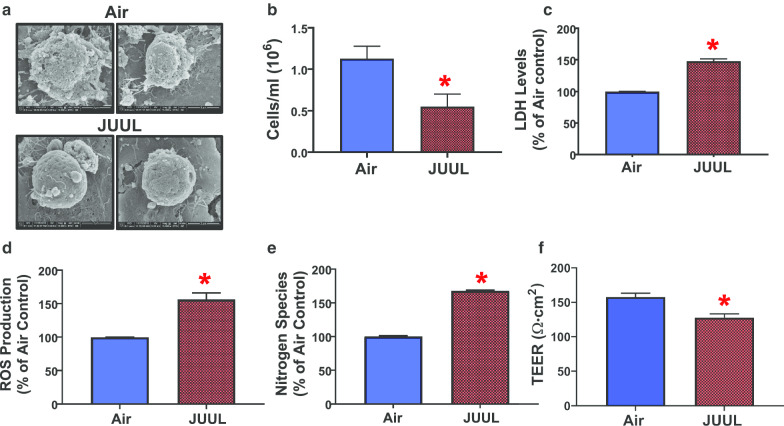
JUUL crème brûlée-flavored aerosols are cytotoxic to BEAS-2B cells. Short-term ALI exposure to JUUL causes (**a**) alterations in cellular surface morphology compared to air controls, as BEAS-2B cells typically have a cobblestone-like appearance as indicated by SEM. Images were taken at 10,000× and 15,000× magnification. **b** JUUL causes a significant decrease in cell viability (n = 8 replicates per group; combined data from three independent experiments each performed in duplicate or triplicate); **c** a significant increase in extracellular release of LDH (n = 3 per group); **d** an increase in extracellular ROS species production (n = 3 per group); **e** an increase in NO species production in BEAS-2B cells compared to air controls (n = 3 per group); **f** and an increase in TEER (n = 3 per group). The student’s t-test was used to compare results between JUUL aerosol-exposed cells and air controls. Data represent the mean ± SEM, *p < 0.05

BEAS-2B cells are a human bronchial epithelial cell line that is widely used in respiratory research [58, 64, 65]. This cell line has been used to develop respiratory ALI models and for the assessment of toxicity of tobacco products, including cigarette smoke [58, 64]. We exposed BEAS-2B cells to crème brûlée-flavored JUUL aerosol. The cellular deposited dose, as measured by the QCM, was 20.8 µg/cm^2^ ± 0.16 (SEM). Typically, BEAS-2B cells have a cobblestone appearance [59]. In comparison to air control cells, JUUL-exposed cells exhibited cell surface morphological changes (Fig. 1a). SEM analysis revealed that structurally, the crème brûlée aerosol-exposed cells were rounder and lacked the cobblestone appearance of the air controls (Fig. 1a). We also observed that JUUL decreased cellular viability (Fig. 1b). This was supported by a 50% increase in LDH activity (Fig. 1c), which indicates that JUUL crème brûlée-flavored aerosol is cytotoxic and causes cellular damage to the plasma membrane. We also observed that crème brûlée-flavored aerosol exposure led to greater than 50% increase in both reactive oxygen species and nitrogen species levels (Fig. 1d, e). Moreover, TEER values were significantly lower in the JUUL exposure group compared to air controls (Fig. 1f), indicating a loss in cellular barrier integrity, which may be related to the increased LDH release and decreased cellular viability (Fig. 1b, c). These findings demonstrate that BEAS-2B cells are sensitive to JUUL crème brûlée-flavored aerosol exposures since only 1 day of exposure at the ALI is cytotoxic, affects oxidative metabolism (ROS/RNS), and tight junction intergrity.

### JUUL crème brûlée-flavored aerosol increases extracellular ROS production in H292 cells

**Fig. 2:**
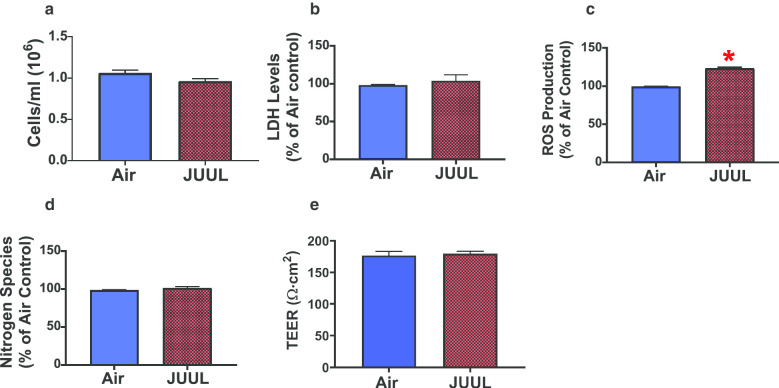
JUUL crème brûlée-flavored aerosol increases extracellular ROS production in H292 cells. 1-day ALI JUUL aerosol exposure had no effect on (**a**) cell viability (n = 3 per group); **b** extracellular LDH release (n = 3 per group); but **c** significantly increased extracellular ROS production (n = 3 per group); while having no effect on (**d**) extracellular NO species production (n = 3 per group); and **e** TEER measurements in H292 cells compared to air controls (n = 3 per group). The Student’s t-test was used to compare results between JUUL aerosol-exposed cells and air controls. Data represent the mean ± SEM, *p < 0.05. Data represent a representative experiment (from three independent experiments) each performed in triplicate (n = 3 per group). See Additional file 1: Figures S1, S2 for the results of the other independent experiments.

H292 cells are a ciliated human bronchial epithelial cell line that is widely used in respiratory research based on its stability and lifespan, in addition to being widely used in toxicological investigations of ALI exposures involving tobacco smoke and e-cig aerosols [66–68]. We exposed H292 cells to crème brûlée-flavored JUUL aerosol, and the cellular deposited dose, as measured by the QCM, was 14.4 µg/cm^2^ ± 5.6 (SEM). In contrast to BEAS-2B cells, H292 cells exhibited no changes in cellular viability or LDH activity (Fig. 2a, b) following exposure to JUUL crème brûlée-flavored aerosols. Further, there was approximately 30% increase in extracellular ROS production (Fig. 2c), but extracellular NO species levels were unchanged (Fig. 2d), and TEER values for H292 cells were similar to those for air controls (Fig. 2e). These results show that of the parameters measured only oxidative stress, measured via ROS levels, is affected in H292 cells exposed for 1 day at the ALI to JUUL crème brûlée-flavored aerosol. Overall, this suggests that JUUL crème brûlée-flavored aerosol exposure is more detrimental to BEAS-2B cells than to H292 cells.

### JUUL crème brûlée-flavored aerosol exposure alters ROS and NO species levels in 246.7 RAW macrophages

**Fig. 3:**
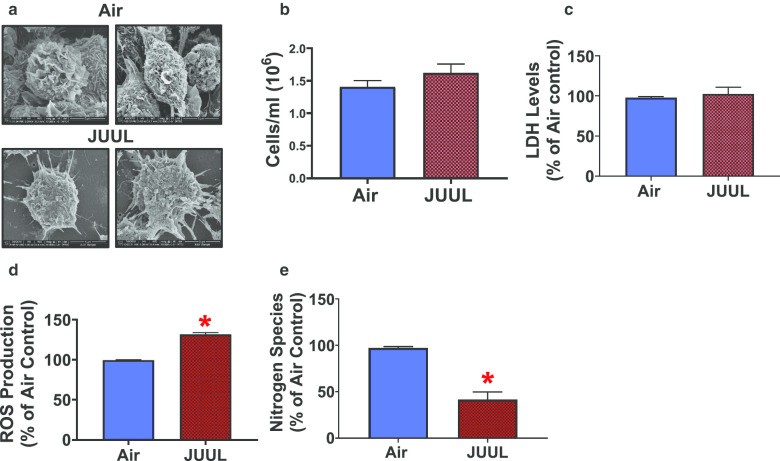
Short-term ALI JUUL aerosol exposure alters extracellular ROS and NO production in RAW 246.7 macrophages. JUUL crème brûlée-flavored aerosol exposure alters (**a**) cellular morphology as indicated by SEM analysis. Images were taken at 10,000× and 15,000× magnification. **b** JUUL crème brûlée-flavored aerosol exposure caused no effect in cellular viability (n = 3 per group); and **c** LDH release in the basal media (n = 3 per group); but significantly **d** increased extracellular ROS production (n = 3 per group); and **e** significantly decreased extacellular NO species production in murine macrophages compared to air controls (n = 3 per group). The Student’s t-test was used to compare results between JUUL aerosol-exposed and air controls. Data represent the mean ± SEM, *p < 0.05. Data represent a representative experiment (from three independent experiments) each performed in triplicate (n = 3 per group). See Additional file 1: Figures S3, S4 for the results of the other independent experiments

To determine the effect of JUUL crème brûlée-flavored aerosol exposures on immune cells, we tested the effects of JUUL on RAW 246.7 macrophages. We exposed RAW macrophages to crème brûlée-flavored JUUL aerosol. The cellular deposited dose, as measured by the QCM, was 15.8 µg/cm^2^ ± 0.17 (SEM). We observed that after only 1-day of exposure, JUUL aerosol changed the morphology of the macrophages, compared to air controls (Fig. 3a). However, there was no effect on cellular viability (Fig. 3b) or LDH extracellular release (Fig. 3c). In addition, JUUL crème brûlée-flavored aerosol significantly increased both extracellular ROS (Fig. 3d) and extracellular NO species production by more than 40% (Fig. 3e). The increased oxidative metabolism (ROS/RNS) observed in RAW 246.7 macrophages after 1 day of exposure to JUUL crème brûlée-flavored aerosol is consistent with macrophage activation.

### JUUL crème brûlée-flavored aerosol dysregulates genes involved in cellular biotransformation, oxidative stress and inflammation in bronchial epithelial cells and macrophages

**Fig. 4:**
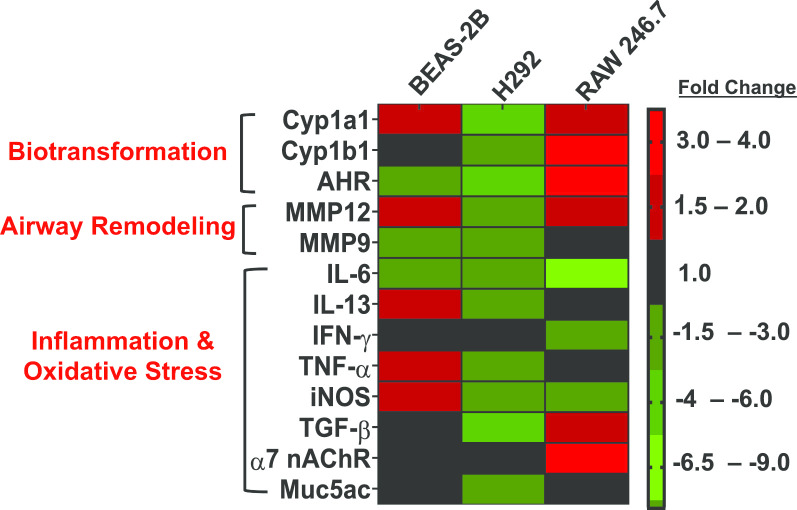
JUUL crème-brûlée aerosols dysregulate genes associated with airway inflammation, and remodeling, biotransformation in human bronchial epithelial cells and murine macrophages. A heatmap displaying cell-type specific expression patterns of dysregulated genes by the JUUL crème brûlée-flavored aerosol exposures. Data is presented as fold-change compared to respective air-control group. For BEAS-2B and RAW 246.7 macrophages: compiled data from two independent experiments each performed in duplicate or triplicate, respectively. H292: data analyzed in triplicate (n = 3 per group). Fold-changes > ± 1.5 were considered significant. Red denotes up-regulation and green denotes down-regulation

To analyze cellular responses at the transcriptional level in all 3 cell lines in response to exposures to JUUL crème brûlée-flavored aerosols, we performed qRT-PCR using a battery of genes that play a role in cellular biotransformation, airway remodeling, oxidative stress and inflammation (Fig. 4). We observed that BEAS-2B cells responded to JUUL crème brûlée-flavored aerosol with increased expression of *CYP1A1, MMP12*, *IL-13, TNF-α and iNOS*, while 3 genes related to aryl hydrocarbon signaling, inflammation and airway remodeling were down-regulated (*AHR, IL-6, MMP-9*). In contrast to results for BEAS-2B cells, JUUL aerosol exposure led to down-regulation of 11 of the 13 genes tested in H292 cells, including *CYP1A1, CYP1B1*, and *AHR* related to biotransformation and aryl hydrocarbon signaling; *MMP-12* and *MMP-9* related to airway remodeling; *IL-6* and *IL-13, TNF-α, iNOS, TGF-β,* and *MUC5AC*, that play a role in inflammatory pathways. Moreover, a total of 6 genes related to biotransformation (*Cyp1a1, Cyp1b1, Ahr*), airway remodeling (*Mmp-12*) and inflammation (*Tgf-β*, and *α7nAChR)*, were upregulated in RAW 246.7 cells. The inflammatory genes *Il-6* and *Ifn-γ* were downregulated in RAW cells, as was the *iNos* gene, which supports the results in Fig. 3e. Our results suggest that expression of various genes related to inflammation, biotransformation and oxidative stress may be cell-specific upon exposure to JUUL aerosols at the ALI.

## Discussion

The combination of widespread use and unknown health effects of new alternative, non-combustion tobacco-related products, delivered by JUUL and related devices, points to a pressing need to investigate respiratory system responses to inhaled JUUL and JUUL-related aerosols. The availability of more than 7700 unique flavors for ENDS, including sweet and fruity flavors [69] is considered the main driver of the recent explosive use of e-cigs among pre-teens, teens and young adults [4–6, 39]. In response, at least in part, to an unexpected public outcry in October 2019, JUUL Labs suspended the sale in the US of their popular flavored pods: crème brûlée, mango, cool mint, and fruit medley [70]. Currently, JUUL Labs only sells in the US menthol- and tobacco-flavored pods [70]. The research reported here on respiratory cell responses to crème brûlée-flavored JUUL aerosol is however, still of paramount importance, as mint, mango and crème brûlée flavored pods are currently offered by JUUL in various European and Asian countries under different names, for example, Vanilla or Royal Crème is equivalent to the former crème brûlée flavor from the US, despite slight differences in e-liquid constituent concentrations [36]. In addition, there are more than three dozen JUUL-like devices still being marketed [71]. Recently, new disposable, pre-filled, one-time use ENDS devices, including Puff Bars, have become commercially available. Puff Bars-type devices are not presently included under the FDA ban that focuses on fruit- and mint-flavored cartridge (or pod)-based ENDS devices [72]. The pre-filled disposable ENDS devices are available in a variety of flavors, including café latte, orange, and mango, and contain either 20 or 50 mg/mL of nicotine salt [72]. This clearly emphasizes the need for further research on flavored nicotine-salt based devices and their aerosols. The goal of the present study was to evaluate the cytotoxic potential of JUUL crème brûlée-flavored aerosol on human bronchial epithelial cell lines (BEAS-2B and H292) and murine macrophages (RAW 246.7) by use of an in vitro ALI system that mimics human exposures.

Results from the present study show that human bronchial epithelial cells and murine macrophages are affected by 1 day of exposure at the ALI to JUUL crème brûlée-flavored aerosol (Figs. 1, 2, 3 and 4). We found that BEAS-2B cells are more sensitive to JUUL exposures than are H292 cells. BEAS-2B cells displayed cytotoxicity, alterations in levels of ROS and RNS, as well as decreased TEER, while H292 cells only showed increased ROS (Figs. 1, 2). Murine macrophages demonstrated signs of activation as evidenced by increased oxidative metabolism, measured by ROS and RNS levels (Fig. 3). These effects also were supported at the molecular level with JUUL crème brûlée-flavored aerosol dysregulating the expression of genes related to biotransformation and oxidative stress, as well as airway remodeling (Fig. 4). Overall, this study shows for the first time that short-term ALI exposures to JUUL aerosol, composed of PG/VG, nicotine salt and crème brûlée flavoring chemicals, can be harmful to bronchial epithelial cells and macrophages.

E-cig device operational settings affects the thermal output, along with the chemical production and the quantity of chemicals that are relased into the aerosol [42, 45]. JUUL devices operate with a lower electrical power output and a lower operating temperature (215 °C) [44, 73] than most e-cig devices. Low levels of carbonyl compounds that we measured in the JUUL aerosol (Table 1) were expected because the JUUL is considered a closed system device (no modifiable operational settings) that produces a lower puff volume due to low atomizer resistance (1.6 Ω), low voltage (3.8 V) and low power (8.1 W) of the device [44, 73] compared to other high-powered open-system e-cig devices [45]. JUUL pods contain either 3% (35 mg/mL) or 5% (59 mg/mL) of nicotine salt [12], that exceeds the levels of freebase nicotine within tobacco cigarettes (2 mg/stick) or other e-liquids (3 to 36 mg/mL) [31, 74]. We found that nicotine concentrations (52.5 mg/mL, Table 1) in JUUL pod liquids were similar to those in previous studies; others, however, have reported nicotine salt concentrations of 60–75 mg/mL within the 5% JUUL pods [31, 44, 73, 75–78], a much higher concentration than advertised by the company (59 mg/mL) [12]. While we did not investigate the levels of benzoic acid in JUUL pod liquids, Pankow et al. [77], reported JUUL pod liquids contain 44.8 mg/mL benzoic acid. We found that JUUL crème brûlée-flavored aerosol contained high levels of benzoic acid (86.9 µg/puff, Table 1). Inhalation of benzoic acid, a respiratory irritant, may aggravate pre-existing respiratory conditions [49]. We also found nicotine levels of 131 μg/puff (Table 1) in JUUL crème brûlée-flavored aerosol, within the range of values reported by other groups (up to ~ 170 µg/puff) [35, 76]. Some studies have reported that nicotine (50–80%) and benzoic acid (~ 80–100%) within a JUUL pod have a high rate of transfer from the liquid to the aerosol [31, 35]. Physiological evidence of high absorption of nicotine salt from JUUL pods was observed in a clinical study by Goniewicz and colleagues [78], who tested urinary cotinine levels, a biomarker of nicotine exposure, in 12–21 year olds who use pod-style devices on a regular basis. The study found that participants who used nicotine salt-containing devices had average urinary cotinine levels of 244.8 ng/mL, which is higher than a previous report of cotinine levels from 13 to 19 year olds (66–132 ng/mL), who regularly smoked tobacco cigarettes [79]. In our study, we found that JUUL crème brûlée aerosol contained low levels of carbonyls, including formaldehyde (0.053 µg/puff, Table 1), which is lower than levels previously reported for JUUL aerosols (0.07–0.50 µg/puff) [73, 76]. The differences in the levels of carbonyls quantified in the aerosols may be due to the use of different methods of quantification (e.g., HPLC vs. GC) or due to the fact that the JUUL aerosols were generated from European pods as opposed to US pods in the present study. Collectively, our results plus others [42, 44, 73, 76] show that JUUL aerosol contains nicotine and harmful chemicals, which may negatively affect the health of lung cells.

In contrast to air control cells, JUUL aerosol-exposed BEAS-2B cells exhibited an altered cell surface structure with a rounder shape and a smoother surface that deviates from their normal cobblestone appearance (Fig. 1a) [59]. We also observed that JUUL significantly decreased cellular viability and led to > 50% increase in LDH release from BEAS-2B cells (Fig. 1b, c), as well as increased ROS/RNS species production (Fig. 1d, e), which indicates a response to oxidative stress. Decreased TEER values (Fig. 1f) in JUUL exposed cells further indicate cellular injury and damage to cell barrier integrity, which is in line with our results from the trypan blue exclusion measures of cell viability and LDH extracellular release which are both complimentary measures of membrane integrity. As mentioned previously, JUUL crème brûlée-flavored pod liquid contain ethyl maltol and vanillin [31]. These flavoring chemicals have been shown to cause changes in cellular physiology including cytotoxicity, oxidative stress, inflammation and barrier dysfunction [27, 28, 30, 80]. Also, these flavorings have been implicated in inducing inflammation and respiratory irritation [29]. Additionally, the effects of these flavoring chemicals on barrier integrity was investigated on another human bronchial epithelial cell line (16-HBE cells). At 20 min post-treatment, ortho-vanillin showed less effect than vanillin, with no loss of barrier function, but increased signs of barrier dysfunction, in comparison to treatment with maltol. Similarly, another study [30] investigated the effects of JUUL and JUUL-like pod aerosols on 16-HBE cells. They reported that JUUL crème brûlée and cool cucumber flavors caused epithelial barrier dysfunction in 16-HBE cells. They also observed a dose-dependent increase per puff in acellular ROS production in several JUUL pod flavors, including crème brûlée, which produced a signifcant ROS increase (after exposure to 5–15 puffs). This is in line with our results for both BEAS-2B and H292 cells. Also in line with our results, Omaiye et al. [31] investigated the toxic response of BEAS-2B cells to JUUL pod aerosol extracts under submerged conditions. They reported that crème brûlée aerosols produced a cytotoxic response at a 10% concentration of JUUL aerosol extract. This finding was strongly correlated to concentrations of nicotine and ethyl maltol; however, there was no change in LDH activity. The variation in LDH results in BEAS-2B cells between Omaiye et al. and our study may be due to differences in exposure methodologies (aerosol extract + submerged cell cultures vs. aerosol + ALI exposures).

In addition, we found differential patterns in the expression of various genes related to inflammation, airway remodeling, and oxidative stress between the 2 bronchial epithelial cell types (Fig. 4). We observed that JUUL exposure led to increased expression of 5 of the 13 genes analyzed, including the biotransformation gene *CYP1A1* (1.6 fold), the inflammatory cytokines *IL-13* (1.9 fold) and *TNF-α* (1.9 fold), as well as increased expression of the airway remodeling gene *MMP-12* (twofold). All of these genes are known to play a role in inflammatory lung disorders, including asthma and COPD [81, 82]. Moreover, nicotine e-cig exposure has been shown to increase production of *IL-13*, and to increase protease expression due to elevated matrix metalloproteinase *MMP-12 *in vivo [83]. Additionally, increased expression of *iNOS* (twofold) in BEAS-2B cells, indicates a potential role in oxidative stress and also supports our results for cellular ROS producton (Fig. 1d). In contrast to BEAS-2B cells, JUUL aerosol-exposed H292 cells exhibited down regulation of 11 of the 13 genes tested (range of: − 1.5 to − 3.4 fold), indicating that JUUL crème brûlée-flavored aerosol may affect gene expression responses in a cell-specific manner. Although we used two human lung epithelial cell lines, BEAS-2B cells are transformed, but non-tumorigenic, whereas H292 cells are tumorigenic. BEAS-2B cells appeared to be more sensitive than H292 cells in terms of toxicity and gene expression (Figs. 1, 2 and 4). It was previously reported that H292 cells are more tolerant to exposures of cigarette smoke extracts than BEAS-2B cells, as evidenced by significant reduction in the viability of BEAS-2B cells, an effect that was only observed at higher doses in H292 cells [84]. In addition to the distinct origins of these 2 cell lines, while non-ciliated airways cells, including BEAS-2B, can undergo mitotic activity leading to cell differentiation, ciliated airway cells, including H292, are at a final differentiated stage [85, 86]. Therefore, differences in cellular differentiation potential and the distinct origin of the two cell lines may play a role in the divergent susceptibility of these 2 cell types to JUUL aerosol exposures, in a similar manner as previously reported with inhalation exposures to naphthalene [86] and NO_2_ [84] in vivo, as well as to cigarette smoke extracts in vitro [84–87].

Recent reports indicate that e-cig aerosols may have detrimental effects on respiratory immunity, involving impairment of macrophage function [88, 89]. We are the first to report responses in a macrophage cell line exposed to JUUL aerosols at the ALI. We observed visual changes in cellular morphology in JUUL-treated cells compared to air control cells. Alterations in cell morphology have been associated with changes in cellular function leading to macrophage activation [90, 91]. ROS production is closely linked to NO generation, and elevated levels of ROS lead to low NO bioavailability [92]. In pathological conditions, overproduction of ROS can occur, leading to oxidative stress [93]. In addition to the observed change in cellular morphology, the modifications in increased oxidative metabolism (ROS/RNS) response that we detected (Fig. 3d, e), support macrophage activation by JUUL crème brûlée-flavored aerosol. Downregulation of the *iNOS* gene (Fig. 4) further supports our observation of low RNS in cellular media. Overall, these in vitro results suggest that in vivo exposures to JUUL aerosols could lead to pulmonary inflammation and oxidative stress. Moreover, the interplay between ROS and NO species production in oxidative stress can play a role in several respiratory conditions, including asthma, COPD and fibrosis [94], and may have important implications for long-term users of ENDS devices, including JUUL.

We found no significant change in cell viability or LDH release in RAW 246.7 macrophages (Fig. 3b, c), indicating that short-term JUUL aerosol is not cytotoxic to those cells. Flavorless e-liquids containing nicotine have been shown to be cytotoxic to macrophages and to induce apoptotic activity in alveolar macrophages [95]. This same study also observed that e-cig vapor condensate (ECVC) exposure lead to increased production of MMP-9, IL-6, TNF-α, CXCL8, and MCP-1. That is in contrast to nicotine-free e-liquid that resulted in lower production of IL-6, CXCL8 and MMP-9. In our study, the inflammatory genes *Il-6* (-eightfold) and *Ifn-γ* (-twofold) were down regulated in RAW cells and we observed no change in expression of *Mmp-9* (Fig. 4). These contrasting sets of results could be due to differences in ENDS aerosol exposure methods (ECVC via submerged culture vs. ALI). In addition, Scott et al. utilized a sub-lethal dose of the ECVC (0.5%) that induced these changes. The upregulation of *Tgf-β* (1.6 fold) could also potentially contribute to the downregulation of inflammatory genes. *Tgf-β,* is a well known pleiotrophic cytokine that plays a role in a variety of phyiological processes, including macrophage differentiation, and control of both inflammatory and anti-inflammatory/regulatory macrophage activity [96]. In addition to the down-regulation of inflammatory genes, and up-regulation of *Tgf-β,* we also observed the up-regulation of 5 other genes: 3 related to biotransformation (*Cyp1a1, Cyp1b1, Ahr*); and 1 each to airway remodeling (*Mmp-12*) and *α7nAChR*, a receptor in which nicotine serves as an agonist. In another study in line with our results, Ween et al., [88] observed a decrease in the secretion of TNFa, IL-1b, IL-6, MIP-1a, MIP-1b, and MCP-1, when THP-1 cells were exposed to to either humectants, 18 mg/mL of nicotine in PG, or various apple-flavored e-liquids, with or without nicotine (18 mg/mL) bubbled in cell culture media. These cytokines all play roles in inflammation as well as macrophage function and recruitment. Collectively, our results along with previous reports suggest that ENDS aerosols, including JUUL, may play a role in reduced immunity through alteration of macrophage function.

In summary, since JUUL pod crème brûlée liquids have a combination of vanillin and ethyl maltol along with other constituents, it is plausible that the combined toxicity of these flavoring chemicals compared to that of the individual components may be enhanced, especially when heated and aerosolized through a JUUL device. Collectively, the combination of ethyl maltol and vanillin flavoring, in addition to high levels of nicotine salts in JUUL, plus PG and VG, may be the cause of some of the toxic responses that we observed (Figs. 1, 2, 3 and 4). Taken together, our data suggest that crème brûlée-flavored JUUL aerosols may affect the respiratory tract differentially depending on the cell type; this may influence pulmonary health, leading to lung injury due to cytotoxicity, increased inflammation and oxidative stress. Although we found that JUUL aerosol exposures at the ALI can induce cell-specific toxicity, increased ROS production was a common biological effect among the 3 cell lines analyzed (Figs. 1, 2 and 3). Other reports examining JUUL aerosol extracts or JUUL aerosol-bubbled media, with submerged cell conditions, also show increased oxidative stress levels, measured via ROS [26, 29, 30, 52, 53]. Therefore, our data and other published reports indicate that increased ROS production may be an early adverse effect induced by nicotine salt-rich JUUL aerosol exposures. While JUUL devices are ‘closed-system’ operating at low heating temperature settings that result in the production of lower levels of harmful chemicals in the aerosols, oxidative stress imbalances may be the key event to initiating long-term pulmonary effects. Overall, these data suggest that ENDS devices are not necessarily ‘*safe*’ and may have harmful implications with prolonged usage.

## Limitations

Our study has limitations, as this study was not designed to tease-out the specific contribution of crème brûlée flavoring or to investigate a flavor or size effect (concentration dependent or comparison with cigarette smoke) on in vitro lung cell toxicity. Our goal was to determine the short-term cellular toxicity of JUUL crème brûlée flavored aerosol on three different cell lines exposed at the ALI. We chose to expose the cells at the ALI to a JUUL aerosol, which includes heated PG/VG, nicotine, benzoic acid and flavoring chemicals, to recreate a “real-world” exposure scenario where ENDS users, particularly JUUL users, are exposed to all of these harmful chemicals at once. We used medical grade compressed air as our only negative control. As stated previously, JUUL is very popular among youth and young adults, with flavored pods being one of the top reasons why never smokers initiate ENDS usage [3–6, 22, 23]. Therefore, ENDS devices are not only used by former smokers but also by youth and young adults who are never smokers. By using only two exposure groups: air controls versus crème brûlée JUUL aerosols, our data indicate that crème brûlée JUUL aerosol is worse than medical grade compressed air when lung cells are exposed in a physiologically relevant ALI exposure model.

## Conclusion

There are thousands of flavors and flavoring combinations of e-liquids on the market with the potential to produce harmful effects when aerosolized through an ENDS device. While more research is needed regarding the potential toxicity associated with inhaling flavoring additives in combination with nicotine salt for future regulation of ENDS products, the present study provides laboratory-based evidence that should be considered regarding regulation of nicotine salt-based products.

## Supplementary information

**Additional file 1: Table S1.** Primer set sequences used in this study. **Figure S1**. JUUL crème brûlée-flavored aerosol increases extracellular ROS production in H292 cells (trial #2). **Figure S2.** JUUL crème brûlée-flavored aerosol increases extracellular ROS production in H292 cells (trial #3). **Figure S3.** Short-term ALI JUUL aerosol exposure alters extracellular ROS and NO production in RAW 246.7 macrophages (trial #2). **Figure S4. **Short-term ALI JUUL aerosol exposure alters extracellular ROS and NO production in RAW 246.7 macrophages (trial #3).

## Data Availability

The datasets analyzed during the current study are available from the corresponding author on reasonable request.

## Abbreviations

ALI: Air–liquid interface
CDC: Centers for Disease Control and Prevention
COPD: Chronic obstructive pulmonary disease
e-cig: Electronic-cigarette
ENDS: Electronic nicotine delivery system
EVALI: E-cigarette or vaping associated lung injury
GC: Gas chromatography
HPLC: High performance liquid chromatography
LC: Liquid chromatography
LDH: Lactate dehydrogenase
LPM: Liters per minute
NO: Nitric oxide
OS: Oxidative stress
PG: Propylene glycol
QCM: Quartz microbalance
ROS: Reactive oxygen species
RNS: Reactive nitrogen species
SEM: Standard error of the mean
SEM: Scanning electron microscopy
TEER: Transepithelial electrical resistance
VG: Vegetable glycerin
