# Supplementary material for: Cell-specific toxicity of short-term JUUL aerosol exposure to human bronchial epithelial cells and murine macrophages exposed at the air–liquid interface
Source: Respir Res. 2020 Oct 17;21:269. doi: 10.1186/s12931-020-01539-1 (PMC7568376; doi:10.1186/s12931-020-01539-1)
Supplement: Supplementary file 1 — Additional file 1: Table S1. Primer set sequences used in this study. Figure S1. JUUL crème brûlée-flavored aerosol increases extracellular ROS production in H292 cells (trial #2). Figure S2. JUUL crème brûlée-flavored aerosol increases extracellular ROS production in H292 cells (trial #3). Figure S3. Short-term ALI JUUL aerosol exposure alters extracellular ROS and NO production in RAW 246.7 macrophages (trial #2). Figure S4. Short-term ALI JUUL aerosol exposure alters extracellular ROS and NO production in RAW 246.7 macrophages (trial #3). [file 12931_2020_1539_MOESM1_ESM.docx]

**Title:** Cell-specific toxicity of short-term JUUL aerosol exposure to human bronchial epithelial cells and murine macrophages exposed at the air-liquid interface

**Additional Information**

**Table S1:** **Primer set sequences used in this study.**

| Gene | Forward primer 5′-3′ | Reverse primer 3′-5 |
| --- | --- | --- |
| *Cyp1a1* | TCGGCCACGGAGTTTCTTC | GGTCAGCATGTGCCCAATCA |
| *Cyp1b1* | AAGTTCTTGAGGCACTGCGAA | GGCCGGTACGTTCTCCAAAT |
| *MMP-12* | GCATGGGCTAGGATTCCACC | CATGAACCGTGAGGATGTTGA |
| *MMP-9* | TGTACCGCTATGGTTACACTCG | GGCAGGGACAGTTGCTTCT |
| *IL-6* | ACTCACCTCTTCAGAACGAATTG | CCATCTTTGGAAGGTTCAGGTTG |
| *IL-13* | AAAGTTCAGGATATGGATTGCGT | ACCTGGCATAGGTGTACTTCT |
| *β-Actin* | GGACCTGACTGACTACCTCAT | CGTAGCACAGCTTCTCCTTAAT |


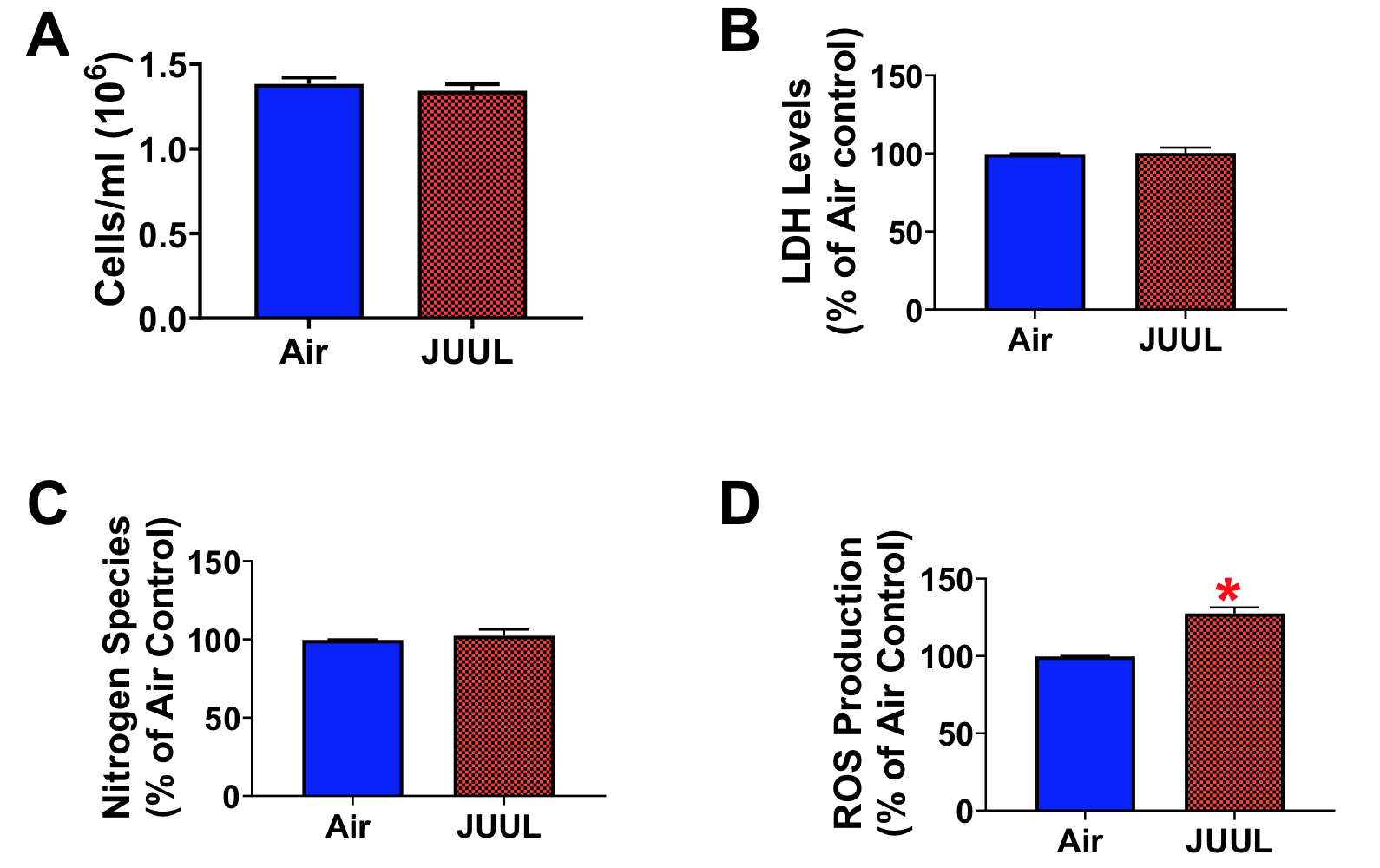


**Figure S1**. **JUUL crème brûlée-flavored aerosol increases extracellular ROS production in H292 cells (trial #2).** 1-day ALI JUUL aerosol exposure had no effect on (A) cell viability (n = 3 per group); (B) extracellular LDH release (n = 3 per group); but (C) significantly increased extracellular ROS production (n = 3 per group) (n1 = 124.4%; n2 = 122.7%; n3 = 135.2%); while having no effect on (D) extracellular NO species production (n = 3 per group); The student’s t-test was used to compare results between JUUL aerosol-exposed cells and air controls. Data represent the mean ± SEM, * p < 0.05.


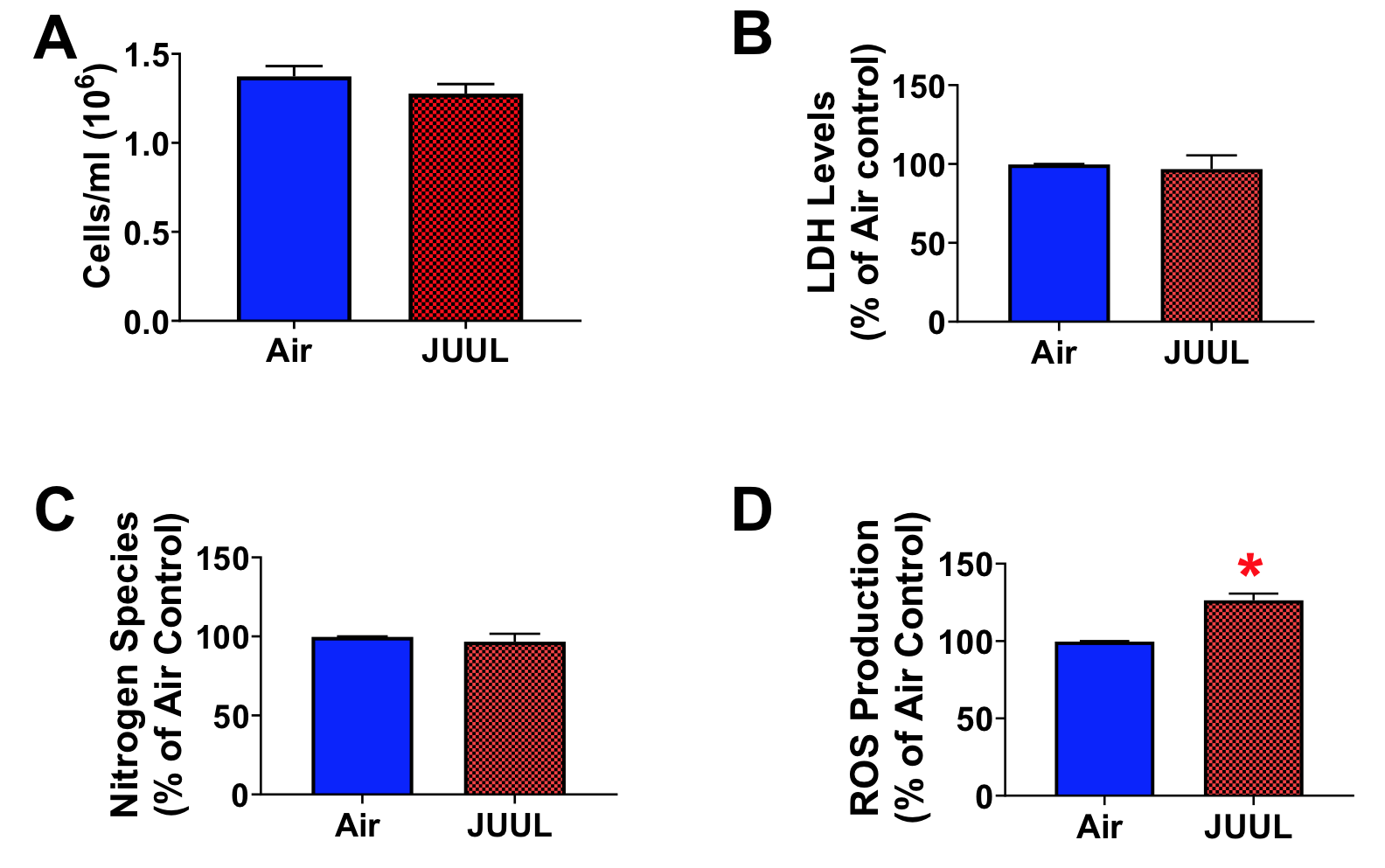


**Figure S2**. **JUUL crème brûlée-flavored aerosol increases extracellular ROS production in H292 cells (trial #3).** 1-day ALI JUUL aerosol exposure had no effect on (A) cell viability (n = 3 per group); (B) extracellular LDH release (n = 3 per group); but (C) significantly increased extracellular ROS production (n = 3 per group) (n1 = 128.2%; n2 = 132.6%; n3 = 118.0%); while having no effect on (D) extracellular NO species production (n = 3 per group); The student’s t-test was used to compare results between JUUL aerosol-exposed cells and air controls. Data represent the mean ± SEM, * p < 0.05.


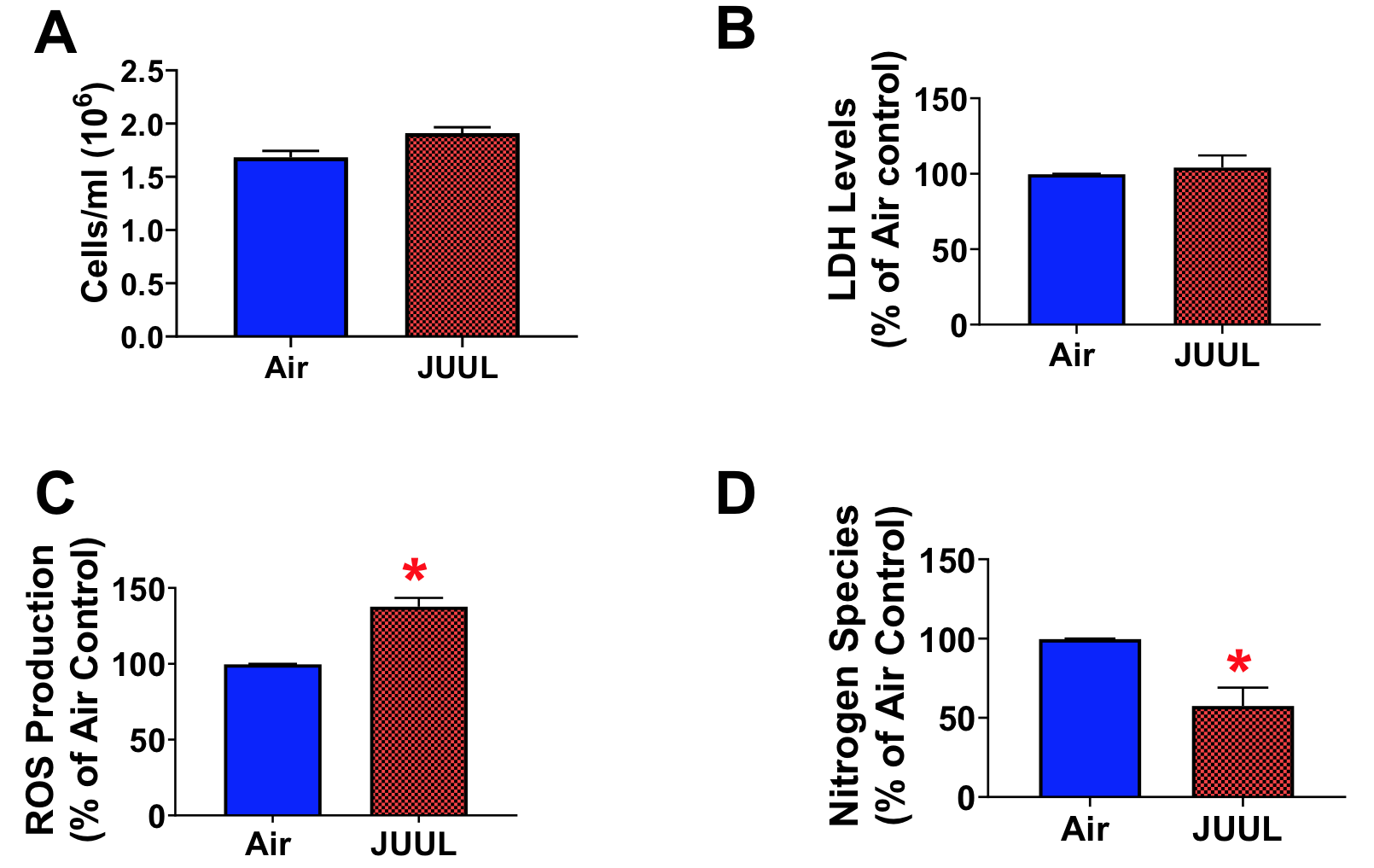


**Figure S3. Short-term ALI JUUL aerosol exposure alters extracellular ROS and NO production in RAW 246.7 macrophages (trial #2).** (A) JUUL crème brûlée-flavored aerosol exposure caused no effect in cellular viability (n = 3 per group); and (B) LDH release in the basal media (n = 3 per group); but significantly (C) increased extracellular ROS production (n = 3 per group) (n1 = 148.2%; n2 = 136.6%; n3 = 128.1%); and (D) significantly decreased extacellular NO species production in murine macrophages compared to air controls (n = 3 per group) (n1 = 65.2%; n2 = 34.5%; n3 = 72.3%). The student’s t-test was used to compare results between JUUL aerosol-exposed and air controls using the student t-test. Data represent the mean ± SEM, * p < 0.05.


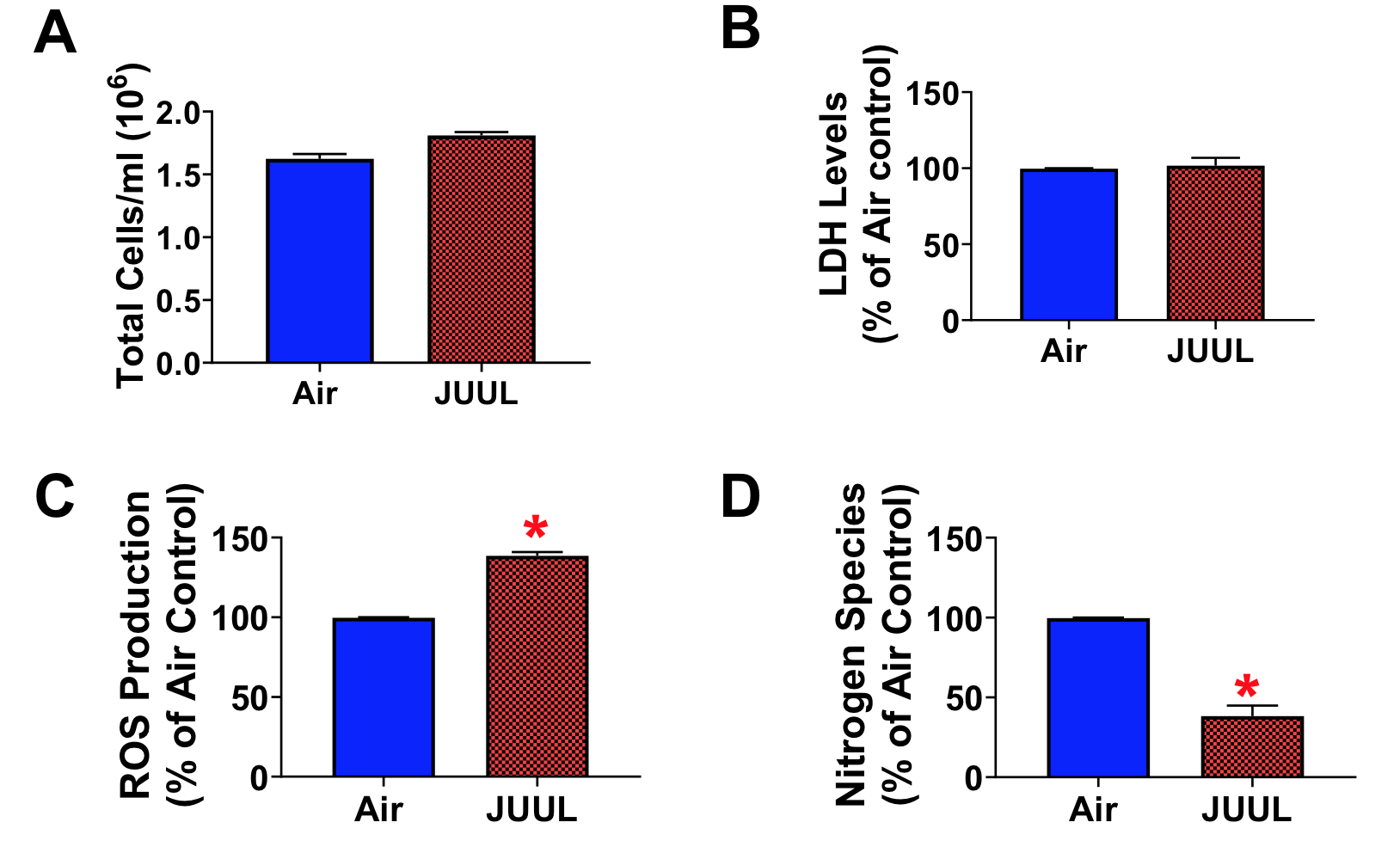


**Figure S4. Short-term ALI JUUL aerosol exposure alters extracellular ROS and NO production in RAW 246.7 macrophages (trial #3).** (A) JUUL crème brûlée-flavored aerosol exposure caused no effect in cellular viability (n = 3 per group); and (B) LDH release in the basal media (n = 3 per group); but significantly (C) increased extracellular ROS production (n = 3 per group) (n1 = 142.8%; n2 = 138.2%; n3 = 134.6%); and (D) significantly decreased extracellular NO species production in murine macrophages compared to air controls (n = 3 per group) (n1 = 49.5%; n2 = 38.6%; n3 = 26.4%). The student’s t-test was used to compare results between JUUL aerosol-exposed and air controls using the student t-test. Data represent the mean ± SEM, * p < 0.05.
